# Clinical Lecture on Cancer of the Tongue

**Published:** 1904-04-02

**Authors:** Harold F. Mole

**Affiliations:** Assistant Surgeon to the Bristol Royal Infirmary.


### Hospital Clinics and Medical Progress.

### CLINICAL LECTURE ON CANCER OF THE TONGUE.

By Harold F. Mole, F.R.C.S., Assistant Surgeon to the Bristol Royal Infirmary.

Cancer occurring in the tongue is that variety known as squamous epithelioma, characterised by the downgrowth of finger-like processes of epithelium from the surface, and the occurrence of cell nests in the deeper parts. Glandular or acinous cancer is not found. The disease is seen most commonly in men after middle life, the tongue being one of the most frequent sites of carcinoma in the male sex. It is very rarely seen in women, the reason for this probably being that prolonged irritation is a frequent exciting cause of cancer, and the male tongue by reason of smoking and spirit drinking is more liable to such irritation. The growth commences as a wart, a crack, or a small ulcer, and there is always early and well-marked infiltration of the base, which, when grasped with the fingers, feels extremely hard and much larger than the appearances only would lead one to expect. In whatever way the growth commences, sooner or later a definite ulcer is produced, which is usually characteristic in its appearance and feel. The surface of the ulcer is irregular, it is rarely very deep, the edges are thickened and everted, and the base infiltrated and hard. Although it may commence in any part of the organ the most common site is the anterior half on the side, often the actual edge of the tongue. From its point of origin it may spread in all directions, but most commonly downwards, involving the mucous membrane of the floor of the mouth, and subsequently the periosteum of the lower jaw, becoming fixed to this bone. Should the growth have commenced more posteriorly the anterior pillar of the fauces may be involved. The growth will not have been present long before the lymphatic glands in the neck become involved by extension along the lymphatic vessels, the cancer cells probably growing along lymphatic spaces. The lymphatic glands most commonly enlarged are the submaxillary, and a gland on the carotid artery, the submental when the disease is very anterior, and the parotid lymphatic gland at the angle of the jaw when the disease is situated more posteriorly. The symptoms of the disease, in addition to the presence of the sore, are first of all a considerable amount of pain, often of a neuralgic character, more or less discharge and salivation, and later inability to protrude the tongue and difficulty in speaking and swallowing, with, as the disease becomes still more advanced, more discharge and dribbling of saliva with occasional hæmorrhages. At the same time the glands in the neck enlarge, become adherent to the surrounding parts, may break down to form cysts containing a clear brownish fluid or the skin may give way over them, leaving a foul excavated ulcer. Should the disease be left untreated the patient dies worn out by continual pain and frequently recurring hæmorrhages, from poisoning by swallowing the discharges from the ulcer, septic pneumonia from inhalation of the same or from inanition owing to inability to swallow a sufficiency of nourishment, or from a combination of some of these causes. A large hæmorrhage from ulceration into the lingual artery sometimes brings about a more sudden termination. The disease seldom spreads beyond the neighbouring lymphatic glands; dissemination in internal organs being a rare occurrence. There will not often arise any great difficulty in the diagnosis of cancer of the tongue. The two conditions which are most likely to be confounded with it are the dental ulcer and the gummatous ulcer. The former is usually quite a small ulcer occurring on the edge of the tongue, due to the constant friction against a sharp or jagged tooth; it is often rather indurated at the base, and, indeed, in the elderly, may, if the source of irritation be not removed, develop into cancer. If the offending tooth is removed or filed smooth and an antiseptic mouth-wash used the ulcer will soon heal; should it not do so it must be treated as if malignant. In differentiating between carcinoma and gumma the following points must be taken into consideration: cancer commences on the surface of the tongue, gumma in the substance; cancer on the edge, gumma on the dorsum; the cancerous ulcer is rather shallow with thick everted edges, the gummatous is deep with thin overhanging edges; in cancer the tongue is often the seat of chronic superficial glossitis, in gumma usually not, although they owe a common origin very frequently to syphilis; the cancer is painful, the gumma not; cancer early interferes with the protrusion of the tongue and speech, gumma does not; cancer occurs at a more advanced age than gumma and the glands in the neck are soon involved in cancer and not so in gumma. When all these points of distinction are present there is no difficulty in diagnosis, but it occasionally happens that the ulcer is not absolutely typical, and the gummatous ulcer may be found on the edge or the cancerous one on the dorsum. Under these doubtful circumstances it is usually advised to put the patient on good large doses of iodide of potassium and await the result. I would strongly advise you against this practice which I believe in many cases to be very misleading and often causing loss of valuable time. In the first place you may require two or three weeks of the treatment before you can decide whether any good will result or not, and secondly in a number of cases the drug has a temporary effect on a cancerous growth, causing it to become a little smaller, possibly by the absorption of some of the round-celled infiltration which is always seen preceding the downgrowth of the epithelial columns and possibly because syphilis is also present, and thirdly the patient will nearly always make out that the medicine is doing him good, and it may be difficult to persuade him of the necessity for an operation even when you have convinced yourself. I have seen a patient taking iodide of potassium to decide whether an ulcer on his tongue was malignant or not when his tongue was so fixed in the floor of his mouth that he could not protrude it half an inch. I should, myself, have taken this as quite sufficient evidence of its cancerous nature without any other sign, for I do not believe gumma ever produces it. If this is a pernicious line of practice what should be done to clear up the doubt? I believe the right thing to do is first to take a scraping from the surface of the ulcer and examine it carefully under the microscope; if it is a gumma only granulation cells will be seen, if cancer often large squamous epithelial cells and sometimes cell nests. If there is any doubt at all about the result of this examination no time should be lost, but with the aid of a little injection of cocaine a small piece should be cut out of the margin of the ulcer and a microscopical examination made. If a suitable piece has been obtained the result will be an infallible guide to the diagnosis and all the objections mentioned under the iodide method avoided. Tertiary ulcerations, other than gummata may be occasionally seen on the tongue and may give rise to difficulty in diagnosis, but attention to the points already mentioned will usually enable one to arrive at a correct conclusion. There are two other ulcers which are rarely found on the tongue, but which may perhaps have to be distinguished from cancer: one is the primary syphilitic sore and the other the tubercular ulcer. With regard to the former the indurated base might suggest a very early stage of epithelioma, but in all probability it would be found in younger subjects and the appearance of secondary symptoms would soon clinch the diagnosis. The only case I have seen occurred on the dorsum of the tongue in the middle line and commenced as a small hard indurated nodule without ulceration. With regard to the tubercular ulcer, in all the cases that I have seen it occurred at the extreme tip of the tongue, was shallow, with sharply defined thin edges, without marked induration, and was very painful and associated with either pulmonary or laryngeal tuberculosis. In two of the cases the ulcer had a peculiar square shape. The question of epithelioma did not arise in any of these cases. Before passing on to the treatment of cancer of the tongue I should like to call your attention to the important connection it has with a disease of the surface of the tongue called chronic superficial glossitis. This affection is found most frequently in men, it is very rare in women, and it occurs most often in those who have had syphilis. It is found in several different forms, but the most important in its bearing on cancer is that in which white thickened opaque patches of epithelium are seen and there may or may not be ridging and furrowing of the surface. Whether this disease can be caused by smoking and other forms of irritation, apart from syphilis, is uncertain, but I am inclined to think that the milder degrees can, but not the more severe and typical ones. In a considerable number of these "white" tongues epithelioma develops after the glossitis has been present for many years. The association of the two diseases is sufficiently common to warrant our assuming that an ulcer of the tongue is epitheliomatous when chronic glossitis is present. Thus, although syphilis is a common cause of both chronic glossitis and gumma these are much less frequently found associated than are chronic glossitis and cancer. As then this affection of the tongue—chronic superficial glossitis—is frequently followed by cancer it behoves us to watch most carefully all cases under our care for the earliest signs of cancerous development, for it is only by the early recognition and complete extirpation that satisfactory and lasting good results can be obtained. A good deal is being made nowadays of the so-called "precancerous" stage. It is obvious that if a cancer is going to develop anywhere there must be a "precancerous" stage, but how can we detect it? We should remove a number of tongues unnecessarily if we excised all that were the seat of chronic glossitis, because they *might* be in a "precancerous" condition. We must have some definite evidence of growth before we can recommend to any patient the mutilation of excision of the tongue. What we wish is a positive diagnosis at the earliest possible moment, not surmises as to whether cancer is going to develop or not. The treatment of cancer of the tongue is removal whenever possible, and as early as possible. It is not always necessary to remove the whole tongue. If the growth is limited to the front part, it will suffice to remove the anterior half, if limited to one edge a lateral half may be removed. An attempt should be made to remove an inch of apparently healthy tissue on all sides of the growth. I cannot describe to you all the many different operations for removal of the tongue, only one or two can be dealt with. Whatever operation is done it is well, when possible, to expend a few days in getting the mouth clean. The teeth should receive special attention, and the mouth should be washed out many times a day with some antiseptic. I prefer carbolic lotion 1 in 100. Of course it is not possible to make the mouth aseptic, but much may be done, and the after treatment and rapidity of healing greatly facilitated by attempts in this direction. In an uncomplicated case, when there is no extensive involvement of the floor of the mouth or of the pillars of the fauces, the best operation is undoubtedly Whitehead's. In this method the tongue is removed with scissors through the mouth, the patient having his head and shoulders well raised or lying over on one side. The tongue is pulled forward with a piece of strong silk threaded through its tip. If one half is to be removed it is slit down the centre, the mucous membrane divided in the floor of the mouth as close to the jaw as possible, the connections with the floor of the mouth then snipped through from before backwards with short snips of the scissors, vessels being caught as divided and the tongue dragged forward with the silk ligature. It is now usually projecting well from the mouth and the mucous membrane may be divided horizontally behind the growth and the muscular part separated partly by snips of the scissors and partly by tearing until the lingual artery is seen towards the middle line and deeply towards the floor of the mouth. It may generally be defined and caught in forceps before division. In this method of operating the bleeding is easily controlled, and what bleeding there is takes place out of the mouth instead of down the pharynx, and so there is no necessity for a preliminary laryngotomy. If the disease involves the mucous membrane of the mouth anteriorly to a large extent with perhaps adhesion to the jaw it will be well to saw through the jaw in the middle line in front, separate the two halves, remove the disease and wire the fragments together. If the disease is situated far back or involves the floor of the mouth extensively on one side it will be well to attack it from the side of the neck by some such incision as Kocher's, the lingual artery being tied and the glands removed through the same incision. When the lymphatic glands are not removed through the same incision as the tongue they must be attacked at a second operation undertaken two or three weeks after the first. Mr. Butlin strongly advocates the removal of the glands whether they can be felt enlarged or not, as he has found that on opening the deep fascia of the neck glands are often found enlarged and infected that could not be felt at all before the operation. He advises that all four groups of glands previously mentioned should be removed with the connective tissue and the submaxillary salivary gland dissected out in one piece if possible. So far I have dealt only with those cases where there was little doubt that the disease could be removed, but unfortunately we are often confronted with very advanced forms of the disease where it is most difficult to decide whether to advise the patient to submit to operation or not. Death from cancer of the tongue is a most horrible one as will have been gathered from some of the symptoms of the final stages already mentioned, and the best rule to follow is that recommended by Mr. Butlin. If the disease can be completely eradicated in the mouth it should be undertaken, no matter what the condition of the glands, for death from glandular involvement is far less dreadful than that from the primary disease. If the disease within the mouth cannot be completely extirpated it should be left alone for recurrence will rapidly ensue and the patient's condition be in no way improved. After the tongue or a portion of it has been removed, the raw surface left should be diminished as far as possible with suitably arranged sutures, those parts not covered in being dusted with iodoform or painted over with Whitehead's varnish, friars' balsam in which the spirit has been replaced by a saturated solution of iodoform in ether. The after treatment is important and one special point is not to allow the patient to consider himself an invalid; let him sit up as soon as he is well round from the anæsthetic and begin washing his mouth out with antiseptic or having it sprayed if he is not able to do this. Get him out of bed the day after operation. I believe I have seen old men die after this operation from being kept in bed too long. Plenty of nourishing liquid food and stimulants must be given by means of a tube fastened to the end of a feeder and passed over the back of the tongue or by means of the nasal tube. Should there be any difficulty in this, nutrient enemata may be required during the first twenty-four hours. You will be surprised how well many patients manage to speak, even when the whole tongue has been removed. When the case is inoperable we must do what we can to relieve the patient's sufferings by means of morphia given by mouth or hypodermically, by cocaine applied locally, and sometimes by division, or, better, excision of a portion of the lingual nerve in the mouth. The mouth must be frequently cleansed by washing out or syringing to prevent the swallowing and inhalation of the discharges. Perhaps the *x*-rays may be of use in the relief of pain as they have been in inoperable carcinoma in other regions.

